# Hospital revisits after paediatric tonsillectomy: a cohort study

**DOI:** 10.1186/s40463-021-00552-8

**Published:** 2022-01-12

**Authors:** Aimy H. L. Tran, Ken L. Chin, Rosemary S. C. Horne, Danny Liew, Joanne Rimmer, Gillian M. Nixon

**Affiliations:** 1 Department of Paediatrics, Monash University and The Ritchie Centre, Hudson Institute of Medical Research, Melbourne, Australia; 2 Melbourne Medical School, University of Melbourne, Melbourne, Australia; 3 Department of Epidemiology and Preventive Medicine, Monash University, Melbourne, Australia; 4 Department of Otolaryngology, Head and Neck Surgery, Monash Health, Melbourne, Australia; 5 Department of Surgery, Monash University, Melbourne, Australia; 6 Melbourne Children’s Sleep Centre, Monash Children’s Hospital, 246 Clayton Road, Victoria, 3168 Australia

**Keywords:** Tonsillectomy, Adenotonsillectomy, Complications, Readmission, Emergency department, Paediatric

## Abstract

**Background:**

Tonsillectomy, with or without adenoidectomy, is the leading reason for paediatric unplanned hospital readmission, some of which are potentially avoidable. Reducing unplanned hospital revisits would improve patient safety and decrease use of healthcare resources. This study aimed to describe the incidence, timing and risk factors for any surgery-related hospital revisits (both emergency presentation and readmission) following paediatric tonsillectomy and adenotonsillectomy in a large state-wide cohort.

**Methods:**

We conducted a population-based cohort study using linked administrative datasets capturing all paediatric tonsillectomy and adenotonsillectomy surgeries performed between 2010 and 2015 in the state of Victoria, Australia. The primary outcome was presentation to the emergency department or hospital readmission within 30-day post-surgery.

**Results:**

Between 2010 and 2015, 46,583 patients underwent 47,054 surgeries. There was a total of 4758 emergency department presentations (10.11% total surgeries) and 2750 readmissions (5.84% total surgeries). Haemorrhage was the most common reason for both revisit types, associated with 33.02% of ED presentations (3.34% total surgeries) and 67.93% of readmissions (3.97% total surgeries). Day 5 post-surgery was the median revisit time for both ED presentations (IQR 3–7) and readmission (IQR 3–8). Predictors of revisit included older age, public and metropolitan hospitals and peri-operative complications during surgery.

**Conclusions:**

Haemorrhage was the most common reason for both emergency department presentation and hospital readmission. The higher risk of revisits associated with older children, surgeries performed in public and metropolitan hospitals, and in patients experiencing peri-operative complications, suggest the need for improved education of postoperative care for caregivers, and avoidance of inappropriate early discharge.

**Graphical Abstract:**

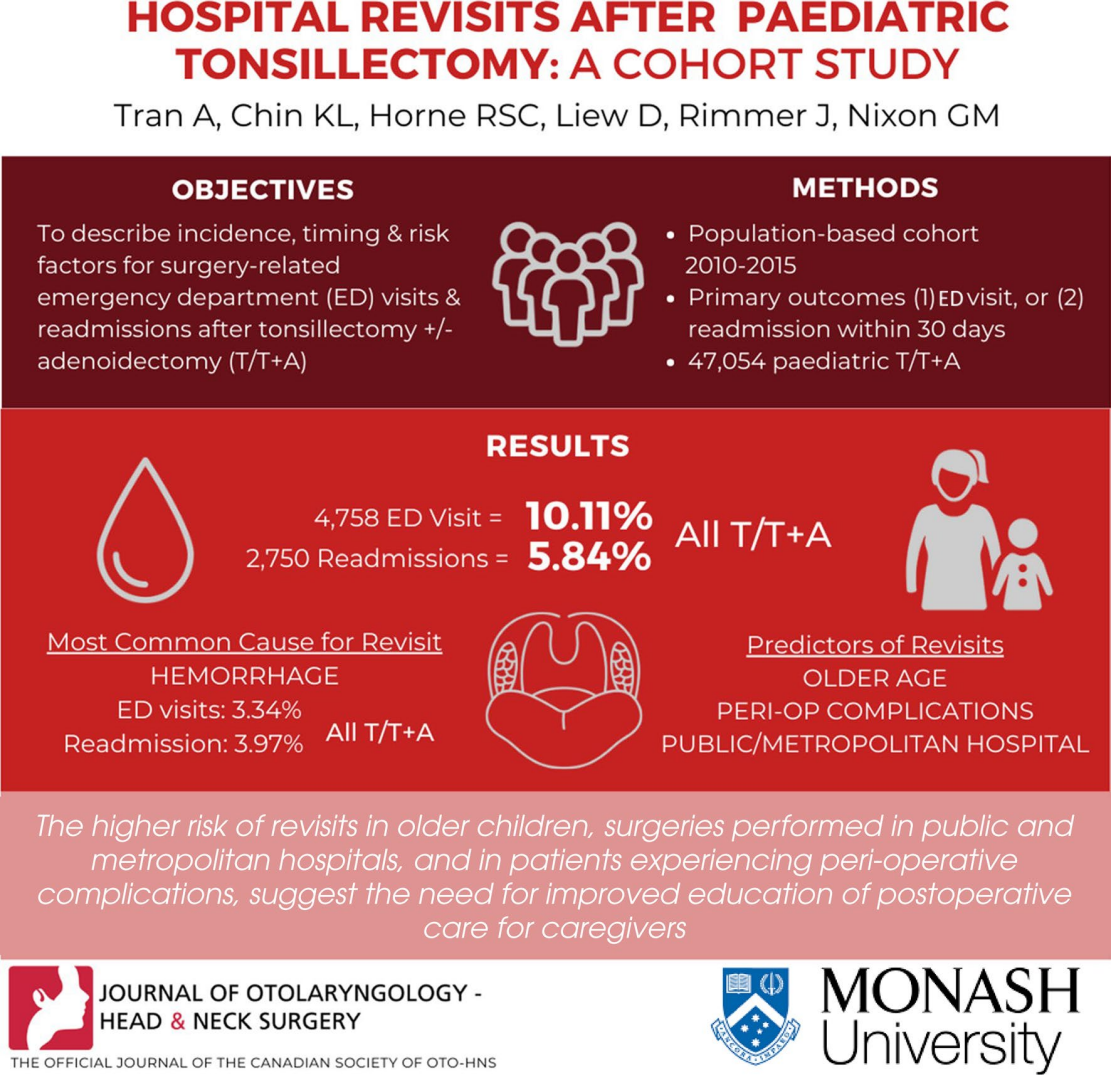

**Supplementary Information:**

The online version contains supplementary material available at 10.1186/s40463-021-00552-8.

## Background

Tonsillectomy, with or without adenoidectomy, is primarily indicated for obstructive sleep apnoea and recurrent tonsillitis and is one of the most common surgeries in children [1]. In the United States (US), more than 289,000 tonsillectomy ambulatory surgeries are performed annually in children under 15 years of age [2]. While these surgeries are relatively safe, post-operative complications can lead to unplanned hospital readmission. Research has identified tonsillectomy to be the most common reason for paediatric unplanned hospital readmission [3], with one study reporting 7.8% of children being readmitted within 30 days after tonsillectomy [4].

Unplanned readmissions result in unnecessary distress to patients and expend healthcare resources [5], costing up to US$17 billion annually in the US [6]. Presentations to the emergency department (ED) after surgery also incur costs. For tonsillectomy, the average cost per patient presenting to the ED is US$1420 [7]. Such revisits represent opportunities for improvement in perioperative care and family education.

Concerted efforts to minimise the risks of complication post-tonsillectomy in children require large, in-depth epidemiological data describing patient characteristics and predictors of such episodes, which are lacking at present. Previous studies have used narrowly defined outcomes, such as only analysing revisits to the same institution, or only reporting readmissions rather than all presentations. We sought to describe the incidence, timing and risk factors for any paediatric tonsillectomy-related hospital revisits (both ED presentation and readmission) in a large, state-wide cohort.

## Methods

### Study design and data sources

According to the last census in 2016, Victoria is Australia’s second most populous state with a total population of 5.93 million people [8]. There are 1.43 million people aged 0–19 years [8], of whom 1.09 million (76%) reside in Melbourne, the state capital [9]. In Victoria, all paediatric tonsillectomy surgeries are performed in the inpatient hospital setting (none in an outpatient setting), with 28% being day case surgery (discharged on post-operative Day 0) [10]. We obtained data capturing patients aged 0–19 years who underwent tonsillectomy, adenoidectomy or adenotonsillectomy between 1 June 2010 and 30 June 2015 across all of Victoria, in both public and private hospitals. We excluded cases that only underwent adenoidectomy, as these have different complication rates to tonsillectomy. Adult patients aged 18 and 19 years were included to capture all paediatric patients because the source data listed age in 5-year bands.

Data on index surgeries and readmissions were drawn from the Victorian Admitted Episodes Dataset (VAED) [11], and data on ED presentations were drawn from the Victorian Emergency Minimum Dataset (VEMD) [12]. Both databases are managed by the Victorian Department of Health. Patient records were linked within and across treating hospitals and then de-identified before being distributed to researchers for analysis.

Hospital revisits were defined as an ED presentation or hospital admission occurring within 30 days of discharge from the index tonsillectomy to any hospital. Surgical indication and revisit reason were recorded using the International Statistical Classification of Diseases and Related Health Problems, Tenth Revision, Australian Modification (ICD-10-AM). Additional file 1 lists the ICD-10-AM codes for each complication category. The VAED allows each episode to have up to 40 diagnosis codes to identify the reasons for readmission, whereas the VEMD allows up to three diagnosis codes to describe the reasons for ED presentation. Although the patients were linked, this coding difference prevents a direct comparison of the reasons for revisit. Therefore, causes of ED presentation and readmission are presented separately. We categorised ICD-10-AM codes into groups of ‘cardiovascular complication’ (e.g. arrhythmias), ‘dehydration’ (e.g. decreased oral intake), ‘pain’, ‘nausea’, ‘haemorrhage’, ‘airway compromise’, ‘upper respiratory complication’ (includes upper respiratory-related infections and inflammation, e.g. infection of the tonsil bed), ‘lower respiratory complication’ (includes lower respiratory-related infections and inflammation), ‘infection’ (infection other than respiratory-related), ‘anaesthesia-related complication’, ‘surgical burn’, ‘unspecified surgical complication’ (e.g. malaise and fatigue, attention to surgical dressings and sutures) and ‘other’ (e.g. ‘sleep disorder, unspecified’). Occurrence of perioperative complications during the index surgery were pre-flagged in the dataset.

Hospital revisits were excluded if the reasons for the revisit were clearly not related to the index surgery (e.g. bone fracture). Transfers to another hospital during the initial admission were not considered revisits.

Variables extracted from the datasets included patient demographic variables (age, sex, area of residence), treating hospital and surgical episode information. Hospitals were de-identified, but each public hospital was allocated a unique code. Private hospitals could not be distinguished from one another (all coded as ‘zero’).

Socioeconomic status was calculated from the Australian Bureau of Statistics’ Socio-Economic Indexes for Areas (SEIFA) using the 2011 Index of Relative Socio-economic Disadvantage [13]. SEIFA uses census data to rank and categorise areas of residence into deciles. We collapsed deciles into quintiles. Residential area of remoteness was determined using the Australian Statistical Geographical Classification—Remoteness Area [14], which is based on road distances from an area to the closest urban area with a population of 1000 or more people (assumed to have some level of healthcare). Furthermore, we reported the Australasian Triage Scale (ATS) categories [15], which are used in Australian EDs to triage cases in order of their clinical urgency.

### Statistical analysis

We conducted all statistical analyses using Stata (release 15.1, StataCorp, Texas, USA). Observations were considered at the surgery level, rather than patient level. To identify risk factors for revisits, all variables described above were included in a multivariable regression model. Statistical significance was defined as two-tailed *P* < 0.05.

## Results

A total of 61,281 paediatric tonsillectomy, adenoidectomy and adenotonsillectomy surgeries were performed in 59,008 patients aged 0–19 years in Victoria between 2010 and 2015. We excluded 14,227 admissions for adenoidectomy alone. Therefore, 47,054 tonsillectomy (with or without adenoidectomy) surgeries were performed in 46,583 patients. Of the 6596 ED presentations, we excluded 1836. Of 3328 readmissions, we excluded 578. These were due to cases undergoing only adenoidectomy, the primary diagnosis at revisit being unrelated to the tonsillectomy or because the revisit was due to a hospital transfer occurring during the index admission. Therefore, there were a total of 4758 ED presentations (10.11% of total surgeries) and 2750 readmissions (5.84% of total surgeries).

Of the ED presentations, 56.85% of cases had a subsequent readmission to hospital. Conversely, while most readmissions were preceded by an ED presentation (the usual pathway), 337 cases (12.25% of readmissions) were directly readmitted without attending an ED. Table 1 summarises the patient demographics, hospital characteristics and types of the index surgeries, ED presentations and readmissions.

**Table 1:** Patient demographics, hospital characteristics and surgical characteristics of tonsillectomy, adenoidectomy and adenotonsillectomy and hospital revisits

	Index surgery		Emergency department presentation		Hospital readmission	
Total	47,054		4758 (10.11% of surgeries)		2750 (5.84% of surgeries)	
*Age*						
0–4 years	16,949	(36.02%)	1447	(30.41%)	705	(25.64%)
5–9 years	16,553	(35.18%)	1330	(27.95%)	693	(25.20%)
10–14 years	6682	(14.2%)	666	(14.00%)	404	(14.69%)
15–19 years	6870	(14.6%)	1315	(27.64%)	948	(34.47%)
*Sex*						
Male	23,624	(50.21%)	2302	(48.38%)	1273	(46.29%)
Female	23,425	(49.78%)	2455	(51.60%)	1477	(53.71%)
Intersex	5	(0.01%)	1	(0.02%)		
*Socioeconomic status (quintiles)*						
1 (lowest SES)	9245	(19.65%)	976	(20.51%)	458	(16.65%)
2	8142	(17.3%)	947	(19.90%)	512	(18.62%)
3	8452	(17.96%)	914	(19.21%)	455	(16.55%)
4	11,476	(24.39%)	1136	(23.88%)	726	(26.40%)
5 (highest SES)	9733	(20.68%)	785	(16.5%)	599	(21.78%)
N/A (overseas)	6	(0.01%)	0	(0%)		
*Area of remoteness*						
Major city	31,150	(66.20%)	3090	(64.94%)	1996	(72.58%)
Inner regional	12,841	(27.29%)	1394	(29.3%)	654	(23.78%)
Outer regional	3038	(6.46%)	272	(5.72%)	99	(3.60%)
Remote or very remote	19	(0.04%)	2	(0.04%)	1	(0.04%)
N/A (overseas)	6	(0.01%)	0	(0%)	0	(0%)
*Surgical indication (index admission)*						
Infective	19,471	(41.38%)	2211	(46.47%)	1400	(51.00%)
Obstructive	14,059	(29.88%)	1290	(27.11%)	683	(24.84%)
Infective and obstructive	12,642	(26.87%)	1200	(25.22%)	633	(23.02%)
Other	882	(1.87%)	57	(1.20%)	34	(1.24%)
*Length of hospital stay during index admission*						
Day case	4755	(10.11%)	436	(9.16%)	247	(8.98%)
Overnight stay	42,299	(89.89%)	4322	(90.84%)	2503	(91.02%)
*Intensive care unit during index admission*						
Yes	285	(0.61%)	60	(1.26%)	47	(1.71%)
No	46,769	(99.39%)	4698	(98.74%)	2703	(98.29%)
*Hospital region*						
Metropolitan	30,773	(65.4%)	3072	(64.56%)	2035	(74.00%)
Regional	16,281	(34.6%)	1686	(35.44%)	715	(26.00%)
*Hospital sector*						
Public	25,132	(53.41%)	4758	(100.00%)	2547	(92.62%)
Private	21,922	(46.59%)	N/A	(0.00%)	203	(7.38%)

Hospital region and hospital sector descriptions for emergency department presentations and readmissions relate to the index surgery the revisit is matched to

There was an average of 985 ED presentations within 30 days of tonsillectomy per year. Table 2 summarises the method of referral, mode of arrival, and the Australasian Triage Scale categories of ED presentation cases. Most presentations to the ED were self-referred, arrived in private transportation and triaged for requiring care within 30–60 min.

**Table 2:** Characteristics of emergency department presentations

	Number of cases (N)	
*Method of referral*		
Self-referred	4392	(92.31%)
Healthcare professional	366	(7.69%)
*Mode of arrival*		
Private transportation	4130	(86.8%)
Road ambulance service	622	(13.07%)
Air ambulance/helicopter	6	(0.13%)
*Australian Triage Scale*		
ATS 1, immediate treatment	5	(0.11%)
ATS 2, treatment within < 10 min	464	(9.75%)
ATS 3, treatment within < 30 min	2276	(47.84%)
ATS 4, treatment within < 60 min	1898	(39.89%)
ATS 5, treatment within < 120 min	115	(2.42%)

*ATS* Australian Triage Scale

There were 236 (4.96%) ED presentations that required transfer to another hospital. The most common reason for transfer was due to a lack of otorhinolaryngologist specialist at the hospital. Table 3 outlines all of the reasons for these transfers.

**Table 3:** Reasons for emergency presentations requiring a transfer to another hospital

	Number of cases (N)	
Total transfer	236	
Intensive Care Unit bed not available	2	(0.85%)
General bed not available	13	(5.51%)
Otorhinolaryngologist not available	177	(75.00%)
Previous patient of the destination hospital	17	(7.20%)
Insurance reasons	3	(1.27%)
Patient preference	2	(0.85%)
Other unspecified reason	22	(9.32%)

Table 4 presents the reasons for ED presentation and for readmission. Haemorrhage was the most common reason for both types of revisits, associated with 33.02% of ED presentations (3.34% total surgeries) and 67.93% of readmissions (3.97% total surgeries). The second most common reason for ED presentation was upper respiratory complications (14.25%), while for readmissions it was dehydration (15.02%).

**Table 4:** Reasons for emergency department presentation and hospital readmission within 30 days of tonsillectomy or adenotonsillectomy

Emergency department presentation			Hospital readmission		
Complications	Percentage (%)	Frequency (N)	Complications	Percentage (%)	Frequency (N)
Haemorrhage	33.02	1571	Haemorrhage	67.93	1868
Unspecified surgical complication	21.02	1000	Dehydration	15.02	413
Upper respiratory complication	14.25	678	Unspecified surgical complication	9.64	265
Infection	13.07	622	Upper respiratory complication	8.87	244
Dehydration	5.07	241	Pain	8.22	226
Other	3.97	189	Infection	7.89	217
Nausea	3.57	170	Nausea	5.78	159
Lower respiratory complication	2.98	142	Lower respiratory complication	4.11	113
Pain	1.85	88	Other	1.67	46
Anaesthesia-related complication	1.51	72	Anaesthesia-related complication	1.38	38
Cardiovascular complication	0.25	12	Cardiovascular complication	0.62	17
Airway compromise	0.06	3	Airway compromise	0.18	7
Surgical burn	0.00	0	Surgical burn	0.07	2

Emergency department presentations and hospital readmissions are mutually exclusive. Overlapping of complications exists within the emergency department presentations because the Victorian Emergency Minimum Dataset allows for up to 3 principal diagnosis codes to be recorded. Similarly, there is overlapping of hospital readmissions as the Victorian Admitted Episodes Dataset allows for up to 40 principal diagnosis codes.

**Fig. 1:**
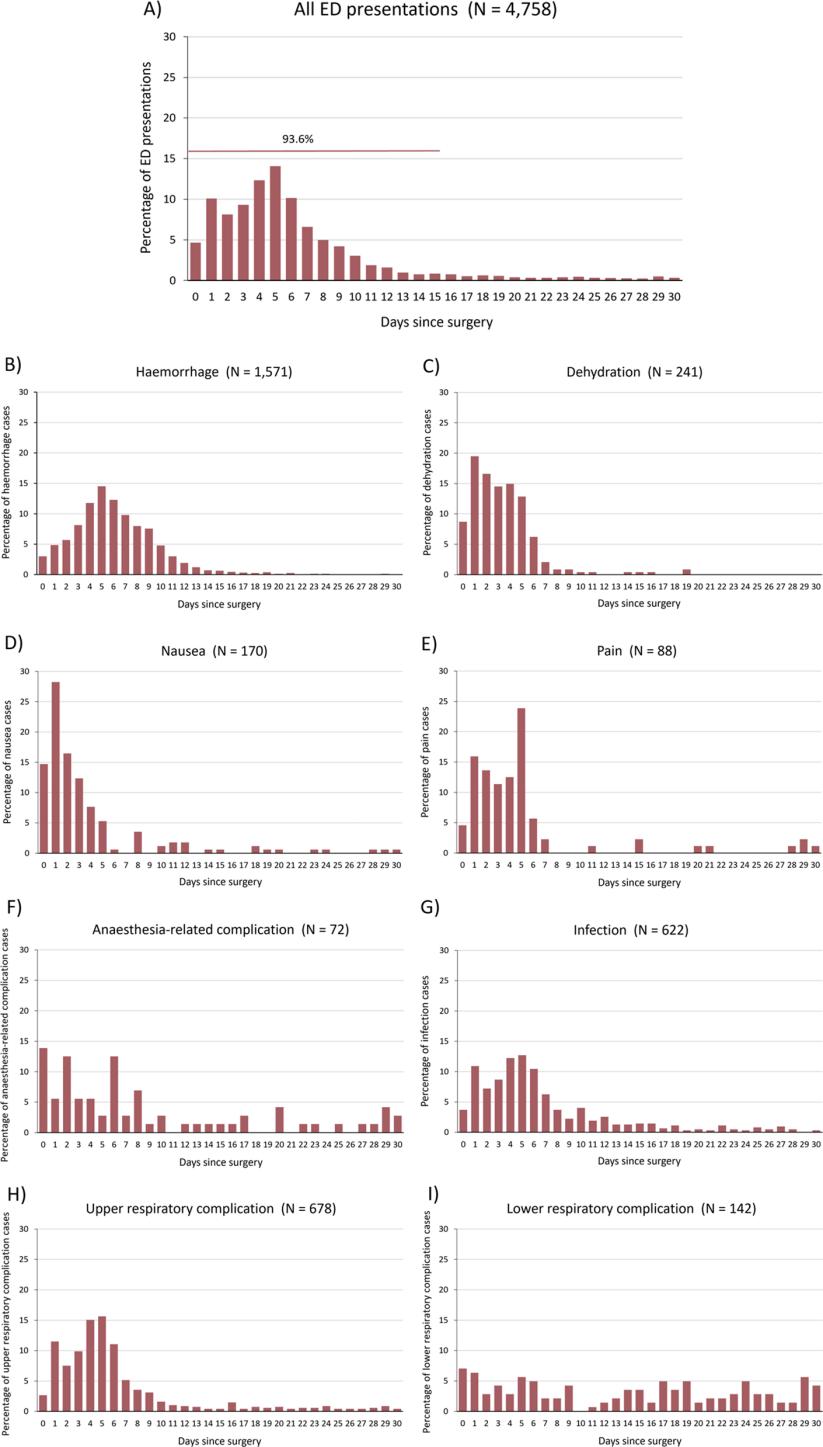
Time distribution to emergency department presentation for **A** All ED presentations; **B** Haemorrhage; **C** Dehydration; **D** Nausea; **E** Pain; **F** Anaesthesia-related complication; **G** Infection; **H** Upper respiratroy complication; **I** Lower respiratroy complication. N.B. Graphs for airway compromise (N = 6) and cardiac complication (N = 14) are not shown as the number of observations are too few. ED = emergency department

**Fig. 2:**
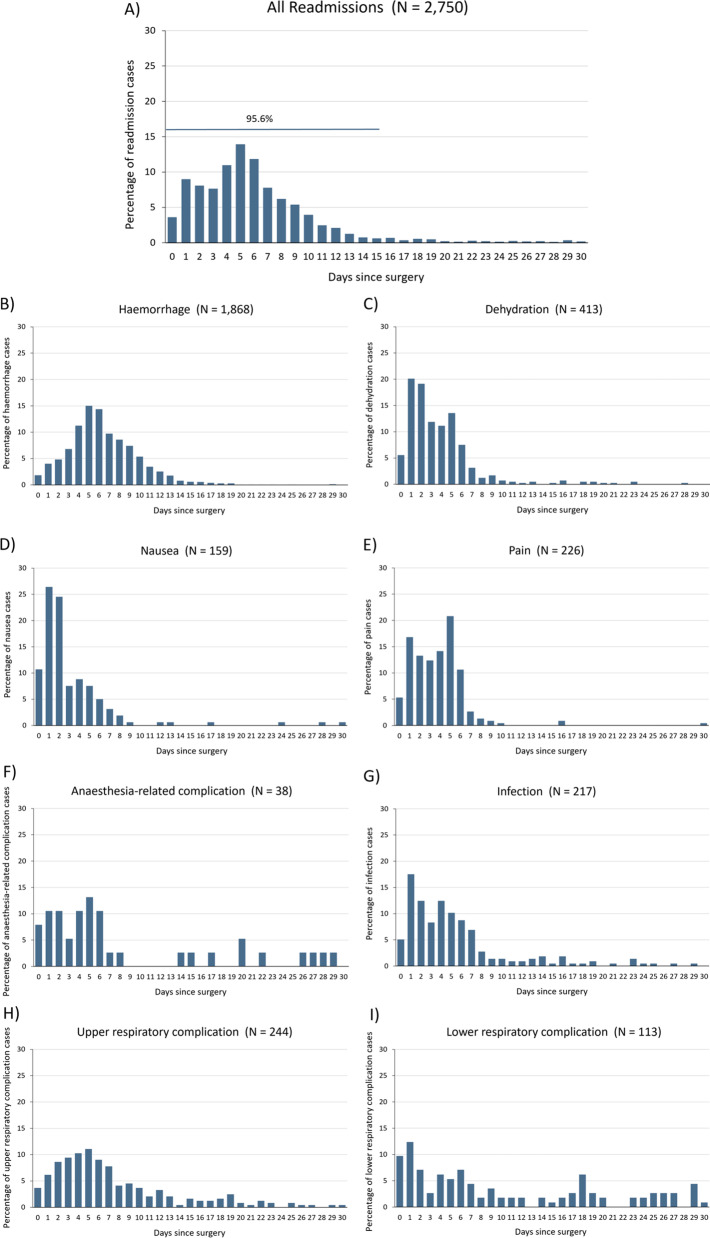
Time distribution to readmissio for **A** All readmissions; **B** Haemorrhage; **C** Dehydration; **D** Nausea; **E** Pain; **F** Anaesthesia-related complication; **G** Infection; **H** Upper respiratory complication; **I** Lower respiratory complication. N.B. Graphs for surgical burn (N = 2), airway compromise (N = 7) and cardiac complication (N = 17) are not shown as the number of observations are too few

Day 5 post-surgery was the median revisit time for both ED presentations (IQR 3–7) (Fig. 1a–i) and readmission (IQR 3–8) (Fig. 2a–i), with 14.08% of ED presentations and 13.93% of readmissions occurring on that day. By Day 1 after surgery, there were 702 ED presentations (14.75%) and 346 readmissions (12.58%), and by Day 15, 4455 of the ED presentations (93.63%) and 2629 of the readmissions (95.60%) had occurred.

Among the ED presentations, most of the revisits involving anaesthesia-related complications and lower respiratory complications occurred on the same day as the surgery, while for dehydration and nausea, most revisits occurred on the day after surgery. Peak presentation for the reasons of infection, haemorrhage, pain and upper respiratory complications occurred at Day 4 or 5. For readmissions, the day after surgery was the most common revisit day for dehydration, nausea, infection and lower respiratory complications. Similar to ED presentations, readmissions involving haemorrhage, pain and upper respiratory complications were most prevalent on Day 5.

### Risk factors for hospital revisits

The risk factors for hospital revisits are presented in Table 5. Although sex was not a significant predictor of hospital visits, this variable was left in the regression models given that it is a key potential confounder related to the severity of sleep disordered breathing and therefore risk of complications [16]. Independent risk factors for both ED presentations and readmissions were the 15–19 age group (compared to the reference 0–4 age group), having surgery at a public or metropolitan hospital, and experiencing a perioperative complication during the index surgery. Both types of revisits also had the protective factor of living in an outer regional area (compared to the reference of living in a major city). Protective factors specific to ED presentations were the 5–9 age group and undergoing surgery for indications other than obstructive or infective. In contrast, risk factors for readmission were the 10–14 age group and lower socioeconomic status (second lowest group compared to the lowest). A protective factor for readmission was living in the inner regional area. The largest predictor for both ED presentation and readmission was the oldest age group of 15–19 years (ED: aOR 2.30, 95% CI 2.09–2.54, P < 0.001; readmission: aOR 3.20, 95% CI 2.84–3.61, P < 0.001).

**Table 5:** Risk factors for hospital revisits

Risk factor	Emergency department presentations			Hospital readmissions		
	aOR	95% CI	*P *value	aOR	95% CI	*P *value
*Age*						
0–4	Reference	–	–	Reference	–	–
5–9	0.89	0.82–0.97	0.01*	0.95	0.85–1.06	0.35
10–14	1.05	0.95–1.17	0.34	1.29	1.12–1.48	< 0.001*
15–19	2.30	2.09–2.54	< 0.001*	3.20	2.84–3.61	< 0.001*
*Sex*						
Male	Reference	–	–	Reference	–	–
Female	0.96	0.90–1.03	0.26	0.99	0.91–1.08	0.78
*Area of remoteness*						
Major city	Reference	–	–	Reference	–	–
Inner regional	1.01	0.91–1.11	0.91	0.85	0.75–0.98	0.02*
Outer regional	0.72	0.61–0.86	< 0.001*	0.53	0.42–0.68	< 0.001*
Remote or very remote	0.79	0.10–5.99	0.82	1.18	0.15–9.04	0.87
*Socioeconomic status*						
1 (lowest SES)	Reference	–	–	Reference	–	–
2	1.06	0.95–1.17	0.29	1.17	1.01–1.34	0.03*
3	0.94	0.85–1.05	0.27	0.97	0.84–1.12	0.66
4	0.98	0.88–1.09	0.73	1.11	0.97–1.27	0.13
5 (highest SES)	0.84	0.75–0.95	0.00	1.08	0.93–1.25	0.29
*Hospital sector*						
Private	Reference	–	–	Reference	–	–
Public	2.04	1.89–2.20	< 0.001*	1.46	1.33–1.60	< 0.001*
*Hospital region*						
Regional	Reference	–	–	Reference	–	–
Metropolitan	1.25	1.13–1.38	< 0.001*	1.31	1.15–1.49	< 0.001*
*Surgical indication*						
Infection	Reference	–	–	Reference	–	–
Obstruction	1.02	0.93–1.11	0.73	0.94	0.84–1.05	0.25
Both	0.94	0.87–1.03	0.20	0.90	0.80–1.00	0.05
Neither	0.73	0.54–0.98	0.04*	0.72	0.50–1.03	0.07
Index surgery complication	1.17	1.01–1.34	0.03*	1.36	1.14–1.61	< 0.001*

*Significant groups, based on a *p* value cut-off of 0.05

Additional file 2 shows the adjusted risk factors for haemorrhage (the most common reason for ED presentations and readmissions). The strongest predictor for both types of revisits for haemorrhage was older aged children of 15–19 years (compared to 0–4 age group).

## Discussion

Our large-scale study provides a complete state-wide analysis of hospital revisits in 47,054 paediatric tonsillectomy surgeries over a 5-year period, and provides the largest review of its kind in an Australian setting. Haemorrhage was the most common reason for revisit, followed by dehydration, “unspecified surgical complication” and upper respiratory complication. The timing of revisits was biphasic, with peaks at Day 1 after surgery (driven by dehydration and nausea) and Day 5 (driven by haemorrhage, pain, infection and upper respiratory complications). Hospital revisits were strongly associated with older age, public and metropolitan treating hospitals and experiencing a peri-operative complication during the index surgical admission.

Our data suggest that the risk of ED presentation within 30 days of surgery was 10.11%, consistent with revisit rates from previous studies ranging from 7.6 to 10.5% in the US [4, 17, 18]. However, our readmission rate (5.84%) was slightly higher than reported ranges of 2.1–3.6% in studies that defined return within 30 days after surgery and included patients 18 years and under [4, 19]. Given the finding of older age as a risk factor for hospital readmissions in our population, the inclusion of patients up to age 19 years in our study may have increased the number of readmissions.

Our analysis showed that haemorrhage was the most common reason for ED presentation (33.02%; 3.34% of total surgeries) and for readmission (67.93%; 3.97% of total surgeries). Previous studies have also shown secondary haemorrhage rates occurring in 0.1–5.0% paediatric tonsillectomy surgeries [5, 20, 21]. However, the literature has reported differing predominant reasons for hospital revisits. While some studies similarly found haemorrhage to be the most common cause [22], others reported dehydration or pain as the predominant reasons [4, 7, 17]. These conflicting results are likely due to how complications are defined and differing readmission guidelines. Nevertheless, our study also found dehydration, nausea and pain to be relatively common reasons for revisit after tonsillectomy surgery. These findings stress the importance of caregiver education on the assessment and management of pain at home, as recommended by the American Academy of Otolaryngology-Head and Neck Surgery guidelines [1]. During the period of this study, providing post-operative information and follow-up was the responsibility of individual surgeons and hospitals. Therefore, this process varied from provider to provider. A recent initiative from the Victorian Department of Health has sought to lead improvements in patient information for tonsillectomy by developing a pre-operative information sheet outlining what to expect in the few days after surgery, a post-operative (discharge) information sheet, a pain management information and plan sheet, and a telephone script to assist clinicians in providing advice when telephoning families on follow-up [23]. A pilot program has documented the success of these measures in reducing readmission after tonsillectomy.

Most ED presentations (93.63%) and readmissions (95.60%) occurred within 15 days of surgery, with 14.08% of presentations to the ED occurring within the first day after surgery. The high proportion of early hospital revisits due to dehydration and nausea suggest that caregiver education of postoperative care can be improved to decrease revisit rates.

Given older age of 15–19 years was the largest predictor of both ED presentation and readmission, strategies to reduce revisits should have a strong focus on these children. Multiple studies have shown an association between older aged children and increasing complication rates, particularly for haemorrhage [17, 24]. No explanation has been presented, but a major reason could be a lower understanding and compliance with post-operative diet, activity and pain management in older children since they have less direct adult supervision. Healthcare workers should therefore stress the importance of management adherence in this age group to patients and families.

We found public and metropolitan treating hospitals to increase the risk of all-cause and haemorrhage-related revisit. This may be due to the model of care in private hospitals, where patients/caregivers have direct access to surgeons for post-operative advice, whereas public patients would be more likely to visit a general practitioner or the ED as a first point of contact. This finding may also be due to the two tertiary hospitals in Victoria, which accept the most complex patients at the highest post-operative risk, being public hospitals situated in metropolitan Melbourne.

Low socioeconomic status was also found to be a risk factor for revisit, which has been supported by many studies, possibly due to increased severity of obstructive sleep apnoea and recurrent tonsillitis [25, 26]. These negative treatment outcomes in low socioeconomic groups may also be worsened due to barriers in effective physician–patient communication, patient education and understanding, and provider bias [27]. Lastly, living in regional Victoria was protective of hospital revisit (compared to metropolitan Victoria). This could be due to the lower number and geographical accessibility of hospitals in regional areas, where patients instead present to a general practitioner.

A key strength of our study is that it comprised a complete capture of all Victorian paediatric tonsillectomy, with or without adenoidectomy, and every related ED presentation and hospital readmission within 30 days of surgery. While strengthening the accuracy of analyses, this also allowed investigation of hospital revisits arriving from different treating hospitals—a limitation of single-centre studies and studies collecting data governed by specific hospitals.

Our use of administrative datasets was a limitation, given that these do not provide granular clinical information. We did not have important information such as surgical technique (e.g. cold steel, diathermy), condition severity, comorbidities, and the quality of post-operative care. Moreover, the nature of the ICD-10 classification system and use of “other” and “unspecified” diagnosis codes impacted on the accuracy of categorising diagnosis codes. This may have explained the high number of infection cases occurring on Day 1 post-surgery, unlikely to represent true surgical wound infection, due to the inclusion of non-specific codes such as “fever” in this category. As suggested by Mitchell et al. [28], who reviewed the use of routinely collected data for analysis of injuries in Australia, we agree with the inclusion of a narrative free text field for admitted episode datasets to provide further detail in these cases. Another limitation was the datasets presenting age in 5-year groupings. To capture the whole paediatric population and particularly the older teenage group when recurrent tonsillitis is prevalent, we included the 15–19 age population in our study. Consequently, some adults aged 18 and 19 have been included in the analyses. This may have added weight to the odds ratio of hospital revisit in the 15–19 age group because the risk of haemorrhage post-tonsillectomy is associated with increased age.

Future studies should incorporate a mixed methods approach to additionally investigate other potential risk factors such as the level of patient understanding of post-operative care and outside-of-hospital factors upon hospital discharge. This is supported by evidence suggesting the main drivers of varying readmission rates are due to the hospital’s patient population and resources of the community [29].

## Conclusions

Our study examined hospital revisits following paediatric tonsillectomy in a large state-wide population. Ten percent of surgeries resulted in a presentation to the ED and five percent were readmitted to hospital. Day 5 post-surgery was the median revisit time for both types of hospital revisit. We found haemorrhage to be the most common reason for both ED presentation and hospital readmission and that the largest predictors of revisit were older age, public and metropolitan treating hospitals, and experiencing a peri-operative complication at the time of surgery. While some of these factors are non-modifiable, there is potential for targeted improvement in how hospitals provide post-operative education to patients and caregivers to reduce hospital revisits, particularly for public and metropolitan hospitals.

## Supplementary Information

**Additional file 1**. ICD-10-AM codes under each complication category. A list of ICD-10-AM codes under each complication category

**Additional file 2**. Risk factors for emergency department presentation and hospital readmission involving haemorrhage. Univariate and multivariable regression analysis of the risk factors for emergency department presentation and hospital readmission involving haemorrhage

## Data Availability

The data that support the findings of this study are available from the Victorian Agency for Health Information but restrictions apply to the availability of these data, which were used under license for the current study, and therefore are not publicly available. Data are however available upon request from the Victorian Agency for Health Information.

## Abbreviations

ATS: Australasian Triage Scale
ED: Emergency department
ICD-10-AM: International Statistical Classification of Diseases and Related Health Problems, Tenth Revision, Australian Modification
SEIFA: Socio-Economic Indexes for Areas
US: United States
VAED: Victorian Admitted Episodes Dataset
VEMD: Victorian Emergency Minimum Dataset
