# Supplementary material for: Hospital revisits after paediatric tonsillectomy: a cohort study
Source: J Otolaryngol Head Neck Surg. 2022 Jan 12;51:1. doi: 10.1186/s40463-021-00552-8 (PMC8756632; doi:10.1186/s40463-021-00552-8)
Supplement: Supplementary file 1 — Additional file 1. ICD-10-AM codes under each complication category. A list of ICD-10-AM codes under each complication category [file 40463_2021_552_MOESM1_ESM.pdf]

SUPPLEMENTAL MATERIAL: ADDITIONAL FILE 1

HOSPITAL REVISITS AFTER PAEDIATRIC TONSILLECTOMY: A COHORT STUDY

Aimy HL Tran, Ken L Chin, Rosemary SC Horne, Danny Liew, Joanne Rimmer, Gillian M Nixon

ICD-10-AM codes under each complication category

ICD-10-AM code	Description
**HAEMORRHAGE**	
R58	Haemorrhage, not elsewhere classified
D67	Hereditary factor IX deficiency
D62	Acute posthaemorrhagic anaemia
T810	Haemorrhage and haematoma complicating a procedure, not elsewhere classified
R041	Haemorrhage from throat
R042	Haemoptysis
R040	Epistaxis
K920	Haematemesis
K921	Melaena
K922	Gastrointestinal haemorrhage, unspecified
T817	Vascular complications following a procedure, not elsewhere classified
I978	Other postprocedural disorders of circulatory system, not elsewhere classified
T811	Shock during or resulting from a procedure, not elsewhere classified
S159	Injury of unspecified blood vessel at neck level
D689	Coagulation defect, unspecified
D680	Von Willebrand's disease
D759	Disease of blood and blood-forming organs, unspecified
D509	Iron deficiency anaemia, unspecified
D508	Other iron deficiency anaemias
D649	Anaemia, unspecified
D500	Iron deficiency anaemia secondary to blood loss (chronic)
I959	Hypotension, unspecified
**CARDIOVASCULAR COMPLICATION**	
I472	Ventricular tachycardia
R000	Tachycardia, unspecified
I471	Supraventricular tachycardia
I499	Cardiac arrhythmia, unspecified
R001	Bradycardia, unspecified
R074	Chest pain, unspecified
**DEHYDRATION**	
E86	Volume depletion
R55	Syncope and collapse

R42	Dizziness and giddiness
R638	Other symptoms and signs concerning food and fluid intake
R633	Feeding difficulties and mismanagement
E870	Hyperosmolality and hypernatraemia
E876	Hypokalaemia
E878	Other disorders of electrolyte and fluid balance, not elsewhere classified
**NAUSEA**	
R11	Nausea and vomiting
**PAIN**	
R13	Dysphagia
R51	Headache
R070	Pain in throat
R529	Pain, unspecified
H920	Otalgia
M436	Torticollis
M542	Cervicalgia
R470	Dysphasia and aphasia
G439	Migraine, unspecified
**ANAESTHESIA-RELATED COMPLICATION**	
R21	Rash and other nonspecific skin eruption
T391	4-Aminophenol derivatives
L270	Generalised skin eruption due to drugs and medicaments
L509	Urticaria, unspecified
T402	Other opioids
D690	Allergic purpura
J950	Tracheostomy malfunction
L500	Allergic urticaria
T806	Other serum reactions
T883	Malignant hyperthermia due to anaesthesia
T885	Other complications of anaesthesia
Y458	Other analgesics and antipyretics causing adverse effects in therapeutic use
Y482	Other and unspecified general anaesthetics causing adverse effects in therapeutic use
Y450	Opioids and related analgesics causing adverse effects in therapeutic use
Y480	Inhaled anaesthetics causing adverse effects in therapeutic use
Y484	Anaesthetic, unspecified causing adverse effects in therapeutic use
Y703	Anaesthesiology devices associated with unintentional events, surgical instruments, materials and devices (including sutures)
T887	Unspecified adverse effect of drug or medicament
T509	Other and unspecified drugs, medicaments and biological substances
L271	Localised skin eruption due to drugs and medicaments
L299	Pruritus, unspecified
T406	Other and unspecified narcotics
Z292	Other prophylactic pharmacotherapy
T856	Mechanical complication of other specified internal prosthetic devices, implants and grafts
T782	Anaphylactic shock, unspecified
F130	Mental and behavioural disorders due to use of sedatives
Z430	Attention to tracheostomy

T784	Allergy, unspecified
T4129	Other specified general anaesthetics
Y400	Penicillins causing adverse effects in therapeutic use
Y579	Drug or medicament, unspecified causing adverse effects in therapeutic use
Y430	Antiallergic and antiemetic drugs causing adverse effects in therapeutic use
Y560	Local antifungal, anti-infective and anti-inflammatory drugs, not elsewhere classified causing adverse effects in therapeutic use
Y401	Cephalosporins and other -lactam antibiotics causing adverse effects in therapeutic use
Y408	Other systemic antibiotics causing adverse effects in therapeutic use
Y455	4-Aminophenol derivatives causing adverse effects in therapeutic use [*note: paracetamol derivative*]
G444	Drug-induced headache, not elsewhere classified
T404	Other synthetic narcotics (poisoning by, adverse effect of and underdosing of)
Z760	Issue of repeat prescription
**INFECTION**	
J36	Peritonsillar abscess
B99	Other and unspecified infectious diseases
A410	Sepsis due to Staphylococcus aureus
A418	Other specified sepsis
B348	Other viral infections of unspecified site
A499	Bacterial infection, unspecified
L089	Local infection of skin and subcutaneous tissue, unspecified
J390	Retropharyngeal and parapharyngeal abscess
R509	Fever, unspecified
R508	Other specified fever
R560	Febrile convulsions
B349	Viral infection, unspecified
I889	Nonspecific lymphadenitis, unspecified
L040	Acute lymphadenitis of face, head and neck
R221	Localised swelling, mass and lump, neck
R591	Generalised enlarged lymph nodes
R227	Localised swelling, mass and lump, multiple sites
H660	Acute suppurative otitis media
H669	Otitis media, unspecified
H653	Chronic mucoid otitis media
H659	Nonsuppurative otitis media, unspecified
H654	Other chronic nonsuppurative otitis media
H609	Otitis externa, unspecified
H699	Eustachian tube disorder, unspecified
H921	Otorrhoea
A4900	Staphylococcal infection, unspecified site
T8578	Infection and inflammatory reaction due to other internal prosthetic devices, implants and grafts
T8141	Wound infection post procedure (excludes Obstetric wound O860)
**AIRWAY COMPROMISE**	
W84	Unspecified threat to breathing
W78	Inhalation of gastric contents
J385	Laryngeal spasm
R090	Asphyxia

J384	Oedema of larynx
R061	Stridor
**UPPER RESPIRATORY COMPLICATION**	
J00	Acute nasopharyngitis [common cold]
A38	Scarlet fever
J069	Acute upper respiratory infection, unspecified
J050	Acute obstructive laryngitis [croup]
J040	Acute laryngitis
R600	Localised oedema
R609	Oedema, unspecified
J359	Chronic disease of tonsils and adenoids, unspecified
J358	Other chronic diseases of tonsils and adenoids
J019	Acute sinusitis, unspecified
J329	Chronic sinusitis, unspecified
J018	Other acute sinusitis
J320	Chronic maxillary sinusitis
J039	Acute tonsillitis, unspecified
J030	Streptococcal tonsillitis
J350	Chronic tonsillitis
J029	Acute pharyngitis, unspecified
J020	Streptococcal pharyngitis
B002	Herpesviral gingivostomatitis and pharyngotonsillitis
B085	Enteroviral vesicular pharyngitis
J343	Hypertrophy of nasal turbinates
R065	Mouth breathing
G4732	Obstructive sleep apnoea syndrome
G4739	Sleep apnoea, other sleep apnoea
G4730	Sleep apnoea, unspecified
**LOWER RESPIRATORY COMPLICATION**	
J46	Status asthmaticus
J22	Unspecified acute lower respiratory infection
R05	Cough
J90	Pleural effusion, not elsewhere classified
J14	Pneumonia due to Haemophilus influenzae
J459	Asthma, unspecified
J450	Predominantly allergic asthma
J958	Other postprocedural respiratory disorders
J690	Pneumonitis due to food and vomit
R068	Other and unspecified abnormalities of breathing
J988	Other specified respiratory disorders
J959	Postprocedural respiratory disorder, unspecified
J683	Other acute and subacute respiratory conditions due to chemicals, gases, fumes and vapours
J984	Other disorders of lung
J960	Acute respiratory failure
J210	Acute bronchiolitis due to respiratory syncytial virus
J219	Acute bronchiolitis, unspecified
J218	Acute bronchiolitis due to other specified organisms

J041	Acute tracheitis
J209	Acute bronchitis, unspecified
J980	Diseases of bronchus, not elsewhere classified
J189	Pneumonia, unspecified
J129	Viral pneumonia, unspecified
J157	Pneumonia due to Mycoplasma pneumoniae
J121	Respiratory syncytial virus pneumonia
J159	Bacterial pneumonia, unspecified
J181	Lobar pneumonia
J180	Bronchopneumonia, unspecified
J123	Human metapneumovirus pneumonia
R062	Wheezing
R060	Dyspnoea
R230	Cyanosis
J981	Pulmonary collapse
J101	Influenza with other respiratory manifestations, other influenza virus identified
J111	Influenza with other respiratory manifestations, virus not identified
J9609	Acute respiratory failure, type unspecified
J9600	Acute respiratory failure, type I
**SURGICAL BURNS**	
T272	Burn of other parts of respiratory tract
T270	Burn of larynx and trachea
T280	Burn of mouth and pharynx
**UNSPECIFIED SURGICAL COMPLICATION**	
R53	Malaise and fatigue
T818	Other complications of procedures, not elsewhere classified
T888	Other specified complications of surgical and medical care, not elsewhere classified
Z488	Other specified surgical follow-up care
T819	Unspecified complication of procedure
Z480	Attention to surgical dressings and sutures
Z452	Adjustment and management of vascular access device
T813	Disruption of operation wound, not elsewhere classified
T889	Complication of surgical and medical care, unspecified
Y848	Other medical procedures as the cause of abnormal reaction, or of later complication, without mention of unintentional events at the time of the procedure
H728	Other perforations of tympanic membrane
H729	Perforation of tympanic membrane, unspecified
E898	Other intraoperative and postprocedural disorders of endocrine and metabolic system
K919	Intraoperative and postprocedural disorder of digestive system, unspecified
K918	Other postprocedural disorders of digestive system, not elsewhere classified
G978	Other intraoperative and postprocedural disorders of nervous system
I978	Other intraoperative and postprocedural complications and disorders of the circulatory system, not elsewhere classified
N998	Other postprocedural disorders of genitourinary system
K913	Postprocedural intestinal obstruction
Z489	Surgical follow-up care, unspecified
M7919	Myalgia, site unspecified
Z4581	Adjustment and management of venous catheter

OTHER	
R69	Unknown and unspecified causes of morbidity
R15	Faecal incontinence
C119	Malignant neoplasm of nasopharynx, unspecified
G470	Disorders of initiating and maintaining sleep [insomnias]
J392	Other diseases of pharynx
Q382	Macroglossia
J348	Other specified disorders of nose and nasal sinuses
Z038	Observation for other suspected diseases and conditions
Z039	Observation for suspected disease or condition, unspecified
Z029	Examination for administrative purposes, unspecified
Z011	Examination of ears and hearing
Z017	Laboratory examination
Z027	Issue of medical certificate
Z049	Examination and observation for unspecified reason
Z099	Follow-up examination after unspecified treatment for other conditions
Z711	Person with feared complaint in whom no diagnosis is made
G479	Sleep disorder, unspecified
G521	Disorders of glossopharyngeal nerve
K590	Constipation
R231	Pallor
Z5188	Other specified medical care
