# Supplementary material for: Hospital revisits after paediatric tonsillectomy: a cohort study
Source: J Otolaryngol Head Neck Surg. 2022 Jan 12;51:1. doi: 10.1186/s40463-021-00552-8 (PMC8756632; doi:10.1186/s40463-021-00552-8)
Supplement: Supplementary file 2 — Additional file 2. Risk factors for emergency department presentation and hospital readmission involving haemorrhage. Univariate and multivariable regression analysis of the risk factors for emergency department presentation and hospital readmission involving haemorrhage [file 40463_2021_552_MOESM2_ESM.pdf]

SUPPLEMENTAL MATERIAL: ADDITIONAL FILE 2

HOSPITAL REVISITS AFTER PAEDIATRIC TONSILLECTOMY: A COHORT STUDY

Aimy HL Tran, Ken L Chin, Rosemary SC Horne, Danny Liew, Joanne Rimmer, Gillian M Nixon

Risk factors for emergency department presentation and hospital readmission involving haemorrhage

	ED presentations			Hospital readmissions		
**Risk Factor**	**aOR**	**95% CI**	**P-value**	**aOR**	**95% CI**	**P-value**
Age						
0-4	Reference	–	–	Reference	–	–
5-9	1.32	1.14 - 1.53	<0.001	1.43	1.24 - 1.66	<0.001
10-14	1.79	1.50 - 2.14	<0.001	2.04	1.72 - 2.41	<0.001
15-19	3.94	3.35 - 4.65	<0.001	4.98	4.28 - 5.81	<0.001
Sex						
Male	Reference	–	–	Reference	–	–
Female	0.92	0.82 - 1.03	0.13	0.91	0.82 - 1.01	0.07
Area of remoteness						
Major city	Reference	–	–	Reference	–	–
Inner regional	0.75	0.63 - 0.89	0.001	0.75	0.63 - 0.88	<0.001
Outer regional	0.40	0.28 - 0.57	<0.001	0.49	0.36 - 0.66	<0.001
Remote or very remote	Removed	–	–	1.85	0.24 - 14.26	0.55
Socioeconomic status						
1 (lowest SES)	Reference	–	–	Reference	–	–
2	0.93	0.77 - 1.11	0.40	1.14	0.96 - 1.35	0.13
3	0.97	0.81 - 1.16	0.71	1.00	0.84 -1.19	0.99
4	0.94	0.80 - 1.12	0.51	1.05	0.89 - 1.23	0.60
5 (highest SES)	0.87	0.72 - 1.05	0.15	1.01	0.84 - 1.20	0.94
Hospital sector						
Private	Reference	–	–	Reference	–	–
Public	1.80	1.59 - 2.03	<0.001	1.52	1.36 - 1.70	<0.001
Hospital region						
Regional	Reference	–	–	Reference	–	–
Metropolitan	1.37	1.16 - 1.62	<0.001	1.41	1.20 - 1.66	<0.001
Surgical indication						
Infection	Reference	–	–	Reference	–	–
Obstruction	1.13	0.98 - 1.31	0.09	1.02	0.89 - 1.16	0.81
Both	1.08	0.93 - 1.24	0.31	0.98	0.86 - 1.12	0.77
Neither	0.77	0.47 - 1.26	0.30	0.89	0.59 - 1.35	0.59
Index surgery complication	0.99	0.78 - 1.27	0.97	1.11	0.89 - 1.38	0.36

* denotes significance at P<0.05. The "remote or very remote" category of area of remoteness was removed in the ED presentation model as were too few numbers in this category that experienced haemorrhage (N = 1).
